# Four decades of transmission of a multidrug-resistant *Mycobacterium tuberculosis* outbreak strain

**DOI:** 10.1038/ncomms8119

**Published:** 2015-05-11

**Authors:** Vegard Eldholm, Johana Monteserin, Adrien Rieux, Beatriz Lopez, Benjamin Sobkowiak, Viviana Ritacco, Francois Balloux

**Affiliations:** 1 Division of Infectious Disease Control, Norwegian Institute of Public Health, Lovisenberggata 8, Oslo 0456, Norway; 2 Instituto Nacional de Enfermedades Infecciosas-ANLIS Carlos Malbrán, Vélez Sarsfield 563, Buenos Aires 1281, Argentina; 3 Department of Genetics, Evolution and Environment, University College London, Darwin Building, Gower Street, London WC1E 6BT, UK

## Abstract

The rise of drug-resistant strains is a major challenge to containing the tuberculosis (TB) pandemic. Yet, little is known about the extent of resistance in early years of chemotherapy and when transmission of resistant strains on a larger scale became a major public health issue. Here we reconstruct the timeline of the acquisition of antimicrobial resistance during a major ongoing outbreak of multidrug-resistant TB in Argentina. We estimate that the progenitor of the outbreak strain acquired resistance to isoniazid, streptomycin and rifampicin by around 1973, indicating continuous circulation of a multidrug-resistant TB strain for four decades. By around 1979 the strain had acquired additional resistance to three more drugs. Our results indicate that *Mycobacterium tuberculosis* (*Mtb*) with extensive resistance profiles circulated 15 years before the outbreak was detected, and about one decade before the earliest documented transmission of *Mtb* strains with such extensive resistance profiles globally.

## Abstract (web summary)

The early origin and evolution of multidrug resistant strains of *Mycobacterium tuberculosis* are poorly understood. Here, the authors perform genomic and phylogenetic analyses of 252 clinical isolates from a tuberculosis outbreak in Argentina and reconstruct the timeline of the acquisition of antibiotic resistance.

## Graphical abstract

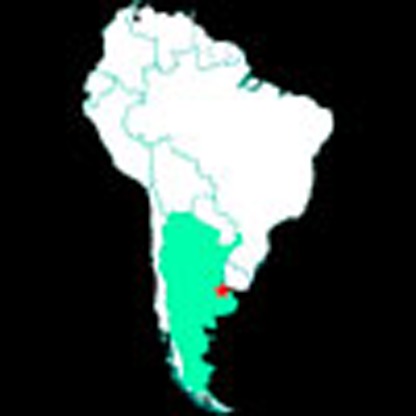

Evolution of antimicrobial resistance has become a global emergency^1^. Recently, studies based on whole-genome sequencing (WGS) of bacterial pathogens have generated important insights into the evolution and spread of drug resistance at global^2^,^3^, continental^4^ and local scales^5^. WGS has also led to novel insights into the evolution of *Mycobacterium tuberculosis (Mtb)* drug resistance. The *Mtb* Beijing lineage is causing a global TB epidemic and has repeatedly been found to be associated with increased levels of drug resistance. WGS has documented the evolution of extensive drug resistance with no apparent loss of transmissibility in this lineage^6^. Interestingly, a recent study applying Bayesian evolutionary analyses to WGS data found that public health weaknesses superimposed on a growing bacterial population rather than selection for antimicrobial resistance has likely caused the expansion of the Beijing lineage^7^.

A number of factors have been suggested to contribute to the increasing rates of drug-resistant TB observed globally, including patient noncompliance with chemotherapy, pharmacokinetic variability and even circulation of counterfeit drugs^8^. However, little is known about the extent of historical drug resistance, and specifically when transmission of resistant strains on a larger scale became a major public health issue. One reason why data on historical transmission of drug-resistant TB are largely missing is the lack of efficient and accurate molecular epidemiological tools before the introduction of IS*6110* restriction fragment length polymorphism (RFLP) typing in the early 1990s (ref. 9). Molecular genotyping has revolutionized TB epidemiology by enabling scientists to identify outbreaks and place clinical isolates in a global phylogenetic context^10^; however, neither the current standard Mycobacterial interspersed repetitive units (MIRU) typing method^11^ nor IS*6110* RFLP^12^ have sufficient resolution to unequivocally detect recent transmission^13^. WGS represents the optimal tool for analysing pathogen microevolution, and has shown great promise in in-depth studies of *Mtb* outbreaks^14^,^15^. However, outbreak analyses are still deeply reliant on labour-intensive epidemiological sleuthing, limiting their application in many regions with high burdens of drug-resistant TB.

To our knowledge, the earliest documented outbreak of polydrug-resistant TB was a school outbreak of isoniazid (INH), streptomycin (STR) and para-aminosalicylic acid triple-resistant TB in 1964–1978 (ref. 16). Outbreaks of multidrug-resistant TB (MDR-TB), resistant to at least INH and rifampicin (RIF), were reported in the United States from 1985 and onwards, often associated with HIV co-infection^17^. Extensively drug-resistant tuberculosis (XDR-TB), defined as MDR-TB with additional resistance to fluoroquinolones (FLQs) and at least one second-line injectable drug, was first defined in 2006 (ref. 18); however, the earliest documented XDR-TB outbreak, identified retrospectively, was actually caused by a strain of *M. bovis* in Spain in 1991 (ref. 19).

A major TB outbreak was first detected in Buenos Aires, Argentina, as a steep upsurge of HIV-related MDR-TB in the early 1990s. Matching IS6110-RFLP patterns documented transmission among HIV-infected patients hospitalized in a referral treatment centre for infectious diseases, in 1992–1995 (ref. 20). Soon, the outbreak strain (the M strain) became the most prevalent MDR *Mtb* genotype countrywide and caused secondary HIV-related transmission in hospitals in nearby districts. The M strain was later found to be responsible for the emergence of MDR-TB in HIV-negative patients with no previous TB treatment^21^, many of whom were health-care workers. In a systematic countrywide survey performed in 2003–2009, the M strain showed a declining trend, but still accounted for 29 and 40% of MDR- and XDR-TB cases, respectively, and as such still represents the largest ongoing outbreak of MDR-TB in Argentina^22^.

Here we utilize WGS to reconstruct the trajectory of drug resistance evolution within the ongoing M outbreak. We sequenced the genomes of 252 clinical isolates belonging to the M outbreak collected between 1996 and 2009 and used a Bayesian phylogenetic approach to date the emergence of resistance-conferring mutations. We find that the progenitor of the M outbreak strain acquired resistance to INH, STR and RIF by around 1973 (95% confidence interval (CI) 1968–1978), thus indicating the continuous circulation of an MDR-TB strain for four decades. Additional resistance to ethambutol (EMB), pyrazinamide (PZA) and kanamycin (KAN) was acquired by around 1979 (95% CI 1975–1983) qualifying the strain for pre-XDR status (*Mtb* resistant to INH and RIF and either FLQs or a second-line injectable agent^23^). This outbreak represents by far the earliest documented transmission of an *Mtb* strain with a pre-XDR phenotype.

## Results

### Phylogenetic analyses and dating the evolution of resistance

**Figure 1:**
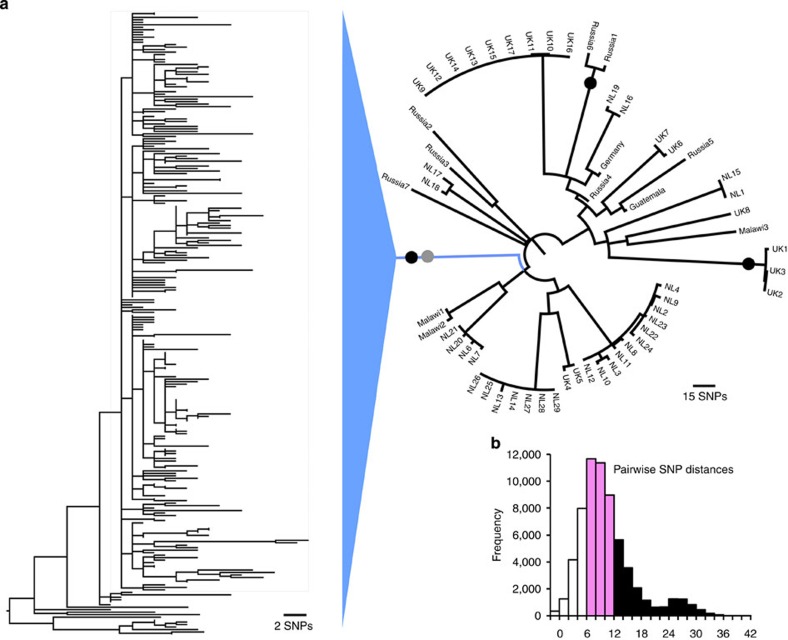
Phylogenetic placement of the M outbreak strains. (**a**) Maximum-likelihood whole-genome SNP phylogeny of sublineage 4.1.2.1 isolates from a global *Mtb* collection. Black dots indicate the acquisition of the *katG* S315T mutation conferring INH resistance. The grey dot indicates the *gidB* V110 frameshift mutation conferring STR resistance. (**b**) Histogram of pairwise SNP distances between M outbreak isolates.

Whole-genome sequence reads from 252 clinical isolates (Supplementary Data 1) were aligned to the H37Rv reference genome. This collection represents all available isolates belonging to the M outbreak on the basis of IS6110 RFLP fingerprinting^22^. On the basis of available information, we believe our collection represents ∼30% of all TB cases caused by the M strain in Argentina (see Methods). After filtering for various quality parameters and removal of single-nucleotide polymorphisms (SNPs) in problematic genomic regions, a total of 509 SNPs were found to separate the 252 isolates with a mean pairwise SNP distance between the isolates of 10.9 (Fig. 1). The M strain could be assigned to sublineage 4.1.2.1 within a recent global *Mtb* SNP phylogeny^24^, as the genomes contained all three characteristic SNPs unique to this sublineage (Supplementary Table 1).

In order to confirm that the M outbreak forms a single monophyletic lineage, we downloaded available sequence reads from isolates belonging to this sublineage, which is widely distributed with sequenced isolates available from the Netherlands, the United Kingdom, Russia, Malawi and Guatemala (Supplementary Data 2). We identified SNPs and indels and created a genome-wide SNP-based maximum-likelihood phylogeny of the 4.1.2.1 sublineage together with our clinical isolates (Fig. 1). The M outbreak isolates form a monophyletic clade separated by at least 80 SNPs from any other 4.1.2.1 isolate (Supplementary Data 3).

To study the temporal evolution of the outbreak, a robust phylogeny, divergence times and evolutionary rates were computed on the basis of the 509 SNPs using BEAST 1.7.4 (ref. 25). The tree was calibrated using sampling dates of the isolates with dates of collection ranging from October 1996 to December 2009. We tested the performance of various demographic models that favoured an exponential growth demographic model. Nucleotide substitution rates and variation among sites were simulated using the general time-reversible substitution model of evolution and a discrete gamma distribution with four rate categories. These analyses resulted in 0.29 (95% CI 0.24–0.34) mutations per genome per year, which is consistent with previous mutation rate estimates for *Mtb* outbreaks^13^,^14^. On the basis of available RFLP data and MDR-TB incidence, the M strain is likely to have circulated in low numbers before slipping into the Muñiz Hospital in Buenos Aires. There it caused a major outbreak among HIV-infected patients associated with very high mortality in 1994–1995 (ref. 20) that also served as a springboard for its expansion beyond the hospital. This scenario is supported by modelling of past population size (Supplementary Fig. 1), which indicates an abrupt increase in bacterial population size in the first half of the 1990s.

**Figure 2:**
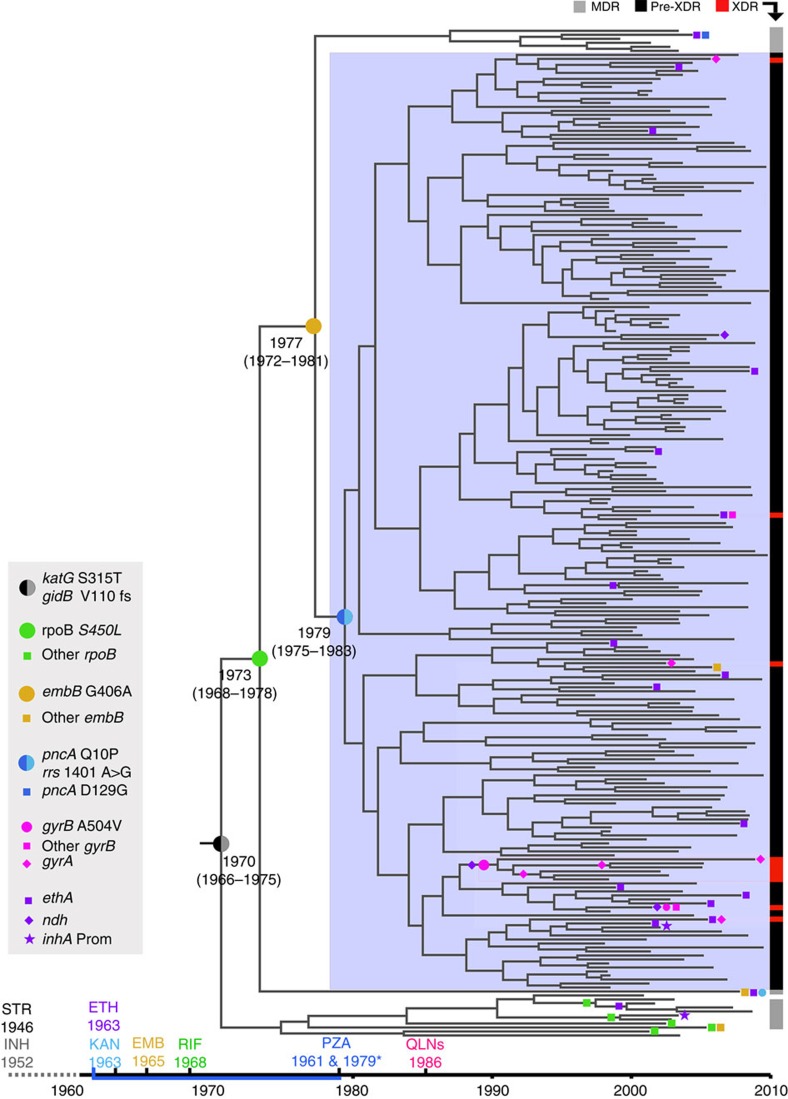
Dated phylogeny of the M outbreak. Resistance mutations are indicated directly on the tree. For resistance mutations that evolved early in the outbreak, the date of acquisition is indicated (95% CI). The timescale at the bottom of the figure illustrates the year of introduction of relevant antibiotics, whereas the vertical bar at the far right illustrates phenotypic drug resistance level as determined by drug susceptibility testing. *PZA was introduced for retreatment cases in 1961 and included in the standard first-line treatment scheme from 1979.

Next, we estimated the age of the most recent common ancestors (MRCAs) of samples carrying resistance mutations that were widespread within the outbreak. These estimates are inherently conservative in the sense that we infer dates for the nodes leading to specific clades harbouring specific resistance mutations, rather than the time of the acquisition of the mutation itself (somewhere on the branch leading to this node). CIs of the inferred dates thus refer to the latest statistically probable dates for emergence of specific mutations. The MRCA of all the isolates was found to have evolved by 1970 (95% CI 1966–1975). Both the *katG* S315T mutation, conferring INH resistance^26^ and a *gidB* V110 frameshift mutation^27^, are common to all the isolates. Resistance to STR is primarily associated with mutations in the three genes *rpsL, rrs* and *gidB*^28^. No *rpsL* or *rrs* mutations were identified in the isolates; however, *gidB* frameshift mutations are well-established determinants of STR resistance^27^. We thus feel confident that the *gidB* V110 frameshift mutation may be responsible for the STR resistance observed in more than 90% of the isolates, even though we are not aware that this specific mutation has been described before. STR and INH were introduced as standard anti-TB drugs in Argentina in 1946 and 1952, respectively^29^. Our results indicate that the MRCA of the outbreak was already resistant to these drugs by 1970 (Fig. 2).

EMB was introduced in 1965 followed by RIF in 1968. With the acquisition of the *rpoB* S450L mutation by 1973 (95% CI 1968–1978), yielding RIF resistance, the strain qualified for MDR status. Our results thus indicate that continuous circulation and transmission of an MDR outbreak strain has taken place for more than four decades and continues to this date.

EMB resistance (*embB* G406A (refs 30, 31)) was acquired by 1977 (95% CI 1972–1981) followed by PZA (*pncA* Q10P (ref. 32)) and KAN (*rrs* 1401A>G (ref. 33)) resistance by 1979 (95% CI 1975–1983), retrospectively qualifying the strain for pre-XDR status. In Argentina, PZA was introduced in 1961 but was restricted to retreatment cases until 1979 when the drug was included in standard short course treatment. Intriguingly, our estimates suggest that PZA resistance evolved the same year as the drug was introduced as part of standardized TB treatment schemes. QLNs were introduced no earlier than 1986 as second-line anti-TB drug. Our estimates show that FLQ resistance had evolved by 1993 (95% CI 1990–1996) but only in a small subset of isolates (Fig. 2), which thus meet the requirements for the XDR status. The dated phylogeny also clearly shows that transmission of FLQ-resistant XDR-TB isolates has occurred.

### Correlation between mutations and phenotypic resistance

There was a good fit between phenotypic drug resistance and predictions on the basis of the presence of putative or known resistance mutations for STR, INH, RIF, PZA and KAN (Table 1 and Supplementary Table 2). The situation for these drugs is fairly simple with a limited number of putative resistance mutations all found in a single gene, and with a single resistance mutation present in almost all resistant strains. The situation for FLQ is somewhat more complicated with multiple candidate mutations in both *gyrA* and *gyrB*. Despite this more complex genetic architecture, the fit between predicted and observed antibiotic resistance remains excellent. Of eight FLQ-resistant isolates, seven had mutations in *gyrA* and/or *gyrB* (Table 1). A *gyrB* A504V (alternatively annotated as A543V) mutation was shared by a cluster of FLQ-resistant isolates (Fig. 2), all of which harboured additional mutations in either *gyrA* or *gyrB*. The A504V mutation was shown to increase the minimal inhibitory concentration (MIC) of ofloxacin moderately from 0.5–1 and 2 μg ml^−1^ in *Mtb* strains H37Rv and Erdman, respectively^34^, and was recently identified alone or in combination with a *gyrA* mutation in FLQ-resistant clinical *Mtb* isolates^35^. A decreased susceptibility to FLQ in isolates harbouring the *gyrB* A504V thus seems likely to have bought these isolates enough time to acquire additional mutations yielding higher levels of FLQ resistance.

**Figure 3:**
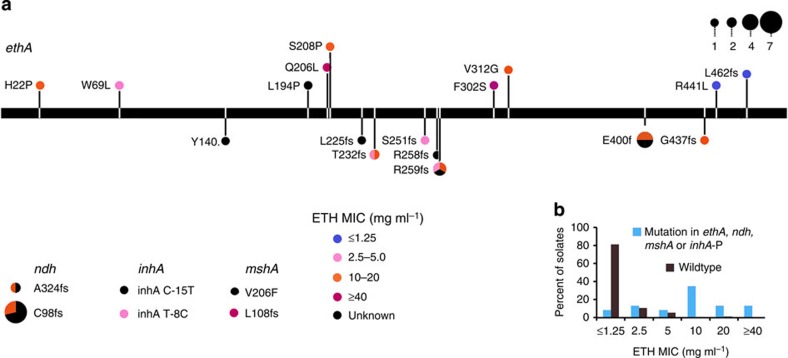
Correlation between ethionamide resistance and mutations in *ethA*, *ndh, mshA* and *inhA* promoter. (**a**) The horizontal bar illustrates the *ethA* gene. Mutations are colour-coded by phenotypic MIC levels observed in the strains harbouring the mutations. Mutations indicated above the bar are nonsynonymous mutations that do not disrupt the reading frame, whereas mutations indicated below the bar result in transcriptional frameshift or the introduction of a premature stop codon. ETH MIC levels in relation to identified mutations in *ndh*, *mshA* and the *inhA* promoter are illustrated separately. Circle size indicates the number of isolates harbouring the individual mutations. (**b**) Summary of MIC levels in wild-type and mutant isolates.

Predicting the resistance to ethionamide (ETH) from genetic data alone is more challenging. Acquisition of mutations in *ethA* is the most common route to ETH resistance; however, mutations in *ndh*, *mshA* and the *inhA* promoter have also been implicated in resistance^36^,^37^,^38^. We considered *inhA* promoter mutations to be more relevant for ETH resistance than INH resistance as all the isolates in the outbreak harbour the widespread *katG* S315T mutation conferring high-level INH resistance^37^. In total, we identified putative ETH resistance mutations in 34 isolates (Fig. 3, Supplementary Data 4). Candidate resistance-conferring mutations were present in all isolates that were resistant to ETH at >5 mg ml^−1^. However, among isolates characterized by low-level resistance to ETH (2.5–5 mg ml^−1^), there were some that did not harbour any obvious candidate resistance mutations. This either suggests that unidentified mutations that cause decreased ETH susceptibility are present, or more likely, reflects the challenges associated with ETH drug susceptibility testing (DST).

### RNA polymerase mutations and fitness compensation

**Figure 4:**
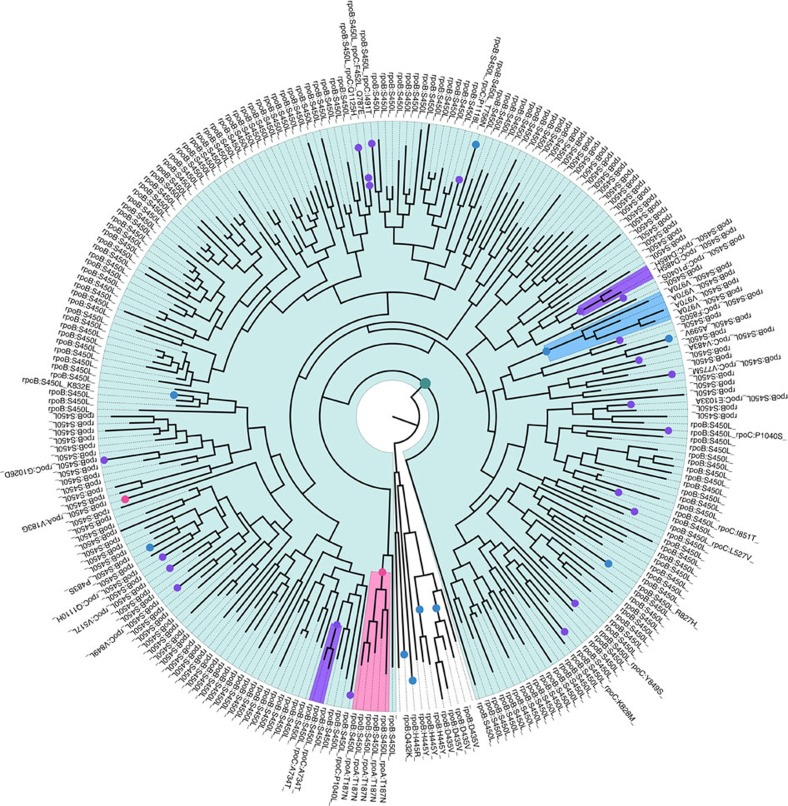
Mutations in RNA polymerase subunits *rpoB*, *rpoC* and *rpoA*. The rpoB *S450L* mutation common to 241 (light-blue-shaded background) of the 252 isolates is indicated by the dark green dot close to the root of the phylogeny. Other mutations in RNA polymerase subunits are indicated with coloured dots (*rpoB*: blue; *rpoC*: magenta; *rpoA*:pink). Mutations common to two or more closely related isolates, indicative of transmission, are indicated by shaded background following the same colour scheme. The amino-acid change caused by each mutation is annotated along the periphery of the phylogeny.

Certain mutations in the RNA polymerase subunits *rpoA* and *rpoC* have been shown to compensate for the fitness loss associated with *rpoB* mutations^39^. To investigate whether we could find evidence for such forces at play in the M outbreak, we mapped all nonsynonymous mutations in all three genes encoding RNA polymerase subunits (Supplementary Data 5) on the phylogeny (Fig. 4). In total, 38 of the 241 isolates harbouring the *rpoB* S450L mutation had acquired secondary mutations in one of the three subunits. Interestingly, despite being frequent, there is little evidence to support an important role of *rpoC* mutations in fitness compensation within this outbreak. In fact, 21 unique *rpoC* mutations were found among 23 isolates (excluding *rpoC* G594E common to all isolates), with only two of these mutations shared by pairs of related isolates. In addition, the mutation *rpoC* P1040S independently evolved in two isolates that do not cluster phylogenetically. This suggests that these *rpoC* mutations do not significantly increase transmission of the isolates harbouring them. We identified two *rpoA* mutations, one in a single isolate, whereas the other (*rpoA* T187N) was common to five clustered isolates. Other mutations at the same codon position were previously identified as high-confidence compensatory mutations^39^, and the transmission of isolates within the M outbreak carrying this mutation would be in line with a fitness-compensatory role. Interestingly, we identified six secondary *rpoB* mutations among the isolates carrying the *rpoB* S450L mutation. One of these, *rpoB* V970A was common to four clustered isolates and could thus also be involved in fitness compensation by the same reasoning as above.

## Discussion

We reconstructed the past demography and timeline of acquisition of antimicrobial drug resistance mutations by generating whole-genome sequences for 252 clinical isolates collected from a single large outbreak in Buenos Aires caused by the M strain. We estimate that the ancestor of the M outbreak qualified for the MDR status by 1973 (95% CI 1968–1978), strongly suggesting the continuous circulation and efficient transmission of a MDR *Mtb* strain over more than four decades, during which time the strain evolved resistance to a number of additional drugs. By 1979 (95% CI 1975–1983) the strain had evolved resistance to six drugs (INH, STR, RIF, EMB, PZA and KAN). As QLNs were introduced in Argentina for treating TB only from 1986, this demonstrates that transmission of *Mtb* resistant to the most efficient drugs available at the time occurred at low rates ∼15 years before the outbreak took off and was detected, and about one decade before the earliest documented transmission of *Mtb* strains with such extensive resistance profiles worldwide.

A perfect correlation between identified resistance mutations and phenotypic *in vitro* resistance is generally not to be expected. Such discrepancy can be explained by a series of causes including within-host bacterial genetic variation, divergent genetic backgrounds of individual clinical isolates or variability in DST performance. As all the isolates in the current study were part of the same outbreak, the genetic diversity is very limited, and variation in genetic backgrounds is thus probably not an important variable. All in all, there was a good fit between identified mutations and resistance phenotype (Table 1).

All *ethA* mutations acquired within the outbreak were nonsynonymous, clearly indicating positive selection and suggesting that most of these mutations might be clinically relevant despite the modest increase in MIC levels associated with some of the mutations. In addition, despite a moderate effect on FLQ susceptibility^34^, the *gyrB* A504V mutation seems to have been important for the development of FLQ resistance in a cluster of XDR-TB isolates within the outbreak (Fig. 2).

We estimated a mutation rate of 0.29 mutations per genome per year over the entire period of the outbreak. Despite this relatively modest rate of evolution, the outbreak strain exhibits an impressive ability to respond to antibiotic challenge by acquiring resistance-conferring mutations. This ability of *Mtb* to rapidly evolving antimicrobial resistance has been observed repeatedly and is intriguing, given the low mutation rate and absence of genetic recombination. In our opinion, this apparent paradox could be best explained by within-host *Mtb* populations being very large, at least in a subset of patients under antibiotic therapy. Such large populations would ensure a constant emergence within infected hosts of very rare resistant variants, which could rapidly increase in frequency following exposure to drugs.

It was recently shown that the evolution of the *Mtb* Beijing lineage in Russia was largely driven by antimicrobial therapy^40^. In the M outbreak, the *rrs* 1401A>G and *pncA* Q10P mutations are the only mutations unique to the large pre-XDR clade and common to all the isolates within it (Supplementary Table 3). We also find that most resistance mutations in individual isolates are descendants of mutations that emerged in the 70s. Together, these findings suggest that antimicrobial resistance has been a major determinant for the successful expansion of the M outbreak strain. We also identified putative fitness-compensatory mutations in genes encoding the RNA polymerase subunits *rpoB*, *rpoC* and *rpoA*. The majority of these were localized in *rpoC*; however, we find little evidence to suggest that these *rpoC* mutations result in increased transmission of the isolates harbouring them.

The efficient transmission and spread of a pre-extensively resistant strain of *Mtb* from 1979 until today in a country with a reasonably well-functioning health system serves as a sombre reminder of the dangers of drug-resistant TB. Although the annual number of cases in this particular outbreak is decreasing^41^, the M outbreak highlights the challenges faced by regions experiencing an increasing burden of drug resistance and the importance of keeping HIV morbidity in check. The patient in which an *Mtb* clone evolved the *pncA* Q10P mutation around 1979 was infected by a strain already resistant to INH, RIF and EMB and was thus probably on a functional PZA monotherapy if the standard first-line drug scheme was followed. We can speculate that the aminoglycoside KAN was administered as a second-line drug following treatment failure, again probably as a functional monotherapy and resulting in the selection of a KAN-resistant *rrs* 1401A>G mutant. This clone became the ancestor of a massive ongoing outbreak of pre-XDR TB. To prevent history from repeating itself over and over again with the evolution and transmission of new clones of drug-resistant TB, the development and implementation of methods for rapid and accurate identification of resistance mutations are dearly needed for appropriate treatment of TB patients, optimally from day one following diagnosis.

## Methods

### Isolate collection

All available isolates belonging to the M outbreak as assessed by IS6110 RFLP were included in the study (see Supplementary Data 1 for a complete list of isolates and their sources). The exact number of lost isolates is not known. No IS6110 RFLP data are available for isolates from before 1992; a freezer accident also contributed significantly to sample loss.

### Genomic analyses

Genomic DNA from clinical *Mtb* isolates was isolated using the CTAB method^9^. Genomic libraries were constructed as described in ref. 42. Briefly, 100–500 ng genomic DNA was used to generate sequencing libraries. DNA was fragmented with NEBNext dsDNA fragmentase for 40 min according to the supplied protocol. Fragmented DNA was purified with Agencourt AMPure beads and Illumina sequencing libraries generated with the High Throughput Library Preparation Kit (KAPA) following the manufacturer's protocol. Individual libraries were indexed with 48-plex NEXTflex barcodes (Bioo Scientific) and sequenced either on the Illumina HiSeq in 100-bp paired-end run mode (244 isolates) or on the MiSeq in 150-bp paired-end mode (eight isolates). Sequencing reads from a global *Mtb* collection and identified as belonging to sublineage 4.1.2.1 were downloaded from the European Nucleotide Archive (accessed 1 February 2015; Supplementary Data 2). Sequencing reads were aligned to the H37Rv genome with SeqMan NGen (DNASTAR). For all isolates, the reads covered >99% of the H37Rv reference genome with a median depth of 108 × coverage. The lowest depth of coverage for any isolate was 47 × . Only SNPs that were sequenced at a depth of at least 8 and present in at least 70% of the reads were included for phylogenetic analyses. SNPs in or within 50 bp distance of regions annotated as PE/PPE genes, mobile elements or repeat regions were excluded from all analyses. SNPs within 10 bp distance from each other were excluded from phylogenetic analyses but were included in analyses of resistance mutations. A maximum-likelihood phylogeny was created in Seaview using a general time reversible (GTR) model with four rate classes^43^. Trees were visualized and edited in Figtree v1.4.2 ( http://tree.bio.ed.ac.uk/software/figtree).

### Phylogenetic evolutionary inferences

Divergence times and evolutionary rates were computed on the basis of an alignment of 509 SNPs from each of the 252 *Mtb* isolates using BEAST 1.7.4 (ref. 25). The XML-input file was manually modified to specify the number of invariant sites.

### Testing for tip-based calibration

As we aimed to calibrate the tree using the dated genomes of *Mtb* only, we started by performing date randomization test to determine whether the temporal and genetic information contained in our data set was sufficient for accurate molecular dating. Ages of the genomes were randomly shuffled 10 times and date-randomized data sets were analysed with BEAST. If the mean estimate of the evolutionary rate or of the time to MRCA (TMRCA) between all isolates obtained from the real data set is not included in any of the 95% highest posterior density intervals of estimates from the date-randomized replicates, then the data set can be considered to have sufficient temporal structure and spread^44^ (Supplementary Note 1, Supplementary Fig. 2). We also investigated the relationship between the ages of the isolates and root-to-tip distances. Branch lengths were estimated using the maximum likelihood algorithm implemented in PhyML^45^ without specifying the age of the isolates and the linear regression of root-to-tip distances against dates of isolation was performed using the Path-O-Gen software (available at http://tree.bio.ed.ac.uk/software/ pathogen/).

### Molecular dating

In BEAST, rates were modelled using the GTR substitution model of evolution and variation among sites was simulated using a discrete gamma distribution with four rate categories. This choice was on the basis of the Bayesian information criteria scores obtained using ModelGenerator v0.85 (ref. 46). We further assumed a lognormal relaxed clock to allow variation in rates among branches in the tree. The tree was calibrated using tip dates only with sample time span ranging from October 1996 to December 2009. Tip dates for each *Mtb* genome were specified in years before the present, with 0 being the youngest sampled strains. We defined flat (that is, uniform) prior distributions for all the nodes in the tree, including the TMRCA of all *Mtb* strains (13–2,000 years old) as well as for the substitution rates (1 × 10^−11^–1 × 10^−5^ substitutions per site per year). We compared the performance of the constant size, logistic growth, expansion growth and exponential growth demographic models based on the Bayes factors calculated from the marginal likelihoods, as recently recommended^47^ (Supplementary Note 2). In addition, to estimate the demographic history of the epidemic without conditioning on a single coalescent model, we independently used the extended Bayesian skyline plot approach integrated in BEAST (Supplementary Fig. 1).

Posterior distributions of parameters, including divergence times and substitution rates, were estimated using Markov chain Monte Carlo (MCMC) sampling. For each analysis we ran four independent chains in which samples were drawn every 5,000 MCMC steps from a total of 50,000,000 steps, after a discarded burn-in of 5,000,000 steps. Convergence to the stationary distribution and sufficient sampling and mixing were checked by inspection of posterior samples (effective sample size >200). Parameter estimation was based on the samples combined from the different chains. The best supported tree was estimated from the combined samples using the maximum clade credibility method implemented in TreeAnnotator.

### Drug susceptibility testing

DST to first-line drugs (INH, RIF, STR, EMB and PZA) was performed in 19 TB network laboratories under regular proficiency testing, according to World Health Organization (WHO) standards^48^. The supranational reference laboratory at the Instituto Nacional de Enfermedades Infecciosas (INEI) ANLIS carried out external quality control, confirmed resistance to first-line drugs and tested susceptibility to second-line drugs (KAN, ETH, amikacin, capreomycin and ofloxacin) following WHO guidelines^49^. For second-line drugs, susceptibility was routinely tested by both the proportion method and MIC. Congruent results classified isolates as either resistant or susceptible, whereas incongruent results were denoted as noninterpretable.

### Ethics statement

This research has been approved by the INEI ANLIS research review board. Microbiological records were handled anonymously so that informed consent was waived.

## Author contributions

V.E., V.R. and F.B. conceived of the study; V.E. and J.M. performed experiments; B.L. and V.R. contributed data; V.E., A.R., B.S., V.R. and F.B. analysed the data; V.E. and F.B. wrote the paper with input from all the authors.

## Additional information

**Accession codes.** Nucleotide data in the form of fastq reads have been deposited in the European Nucleotide Archive database under study accession code PRJEB7669.

**How to cite this article:** Eldholm, V. *et al*. Four decades of transmission of a multidrug-resistant *Mycobacterium tuberculosis* outbreak strain. *Nat. Commun.* 6:7119 doi: 10.1038/ncomms8119 (2015).

## Supplementary Material

Supplementary Information Supplementary Figures 1-2, Supplementary Tables 1-3, Supplementary Notes 1-2, and Supplementary References

Supplementary Data 1 M outbreak isolates information.

Supplementary Data 2 Global collection of sublineage 4.1.2.1 isolates from Coll *et al*. (doi:10.1038/ncomms5812).

Supplementary Data 3 Unique variants common to M outbreak isolates but absent in a global collection of sublineage 4.1.2.1 isolates from Coll *et al*. (doi:10.1038/ncomms5812).

Supplementary Data 4 Ethionamide MIC results and putative resistance mutations.

Supplementary Data 5 mutations in *rpoB*, *rpoC* and *rpoA*.

## Figures and Tables

**Table 1:** Correlation between phenotypic drug susceptibility of clinical isolates and identified putative resistance mutations*.

**Antibiotic**	**Mutation 1**	**Mutation 2**	**Resistant**	**Susceptible**	**Not determined**
STR	***gid*** **V110 fs**		215	22	15
	No mutation		0	0	0
	Total		215	22	15
INH	***katG*** **S315T**		252	0	0
	No mutation		0	0	0
	Total		252	0	0
RIF	***rpoB*** **S450L**		241	0	0
	*rpoB* Q432K		0	1	0
	*rpoB* D435V		4	0	0
	*rpoB* H445Y		3	0	0
	*rpoB* H445R		1	0	0
	No mutation		0	2	0
	Total		249	3	0
EMB	***embB*** **G406A**		150	74	16
	*embB* M306I		1	0	0
	*embB* M306V		1	0	0
	No mutation		1	9	0
	Total		153	83	16
PZA	***pncA*****Q10P**		195	18	21
	*pncA* D129G		0	1	0
	No mutation		1	15	1
	Total		196	34	22
KAN	**rrs 1401A>G**		174	22	39
	No mutation		1	14	2
	Total		175	36	41
FLQ	*gyrB* A504V	*gyrB* R446S	0	0	1
	*gyrB* A504V	*gyrA* A90V	2	0	0
	*gyrB* A504V	*gyrA* D94G	1	0	2
	*gyrB* A504V	*gyrA* L105R	1	0	0
	*gyrA* D94N		1	0	0
	*gyrB* D461V		1	0	0
	*gyrA* R292G		1	0	0
	*gyrB* R446C		0	1	0
	*gyrA* A90V		0	0	1
	No mutation		1	143	96
	Total		8	144	100

EMB, ethambutol; FLQ, fluoroquinolone; INH, isoniazid; KAN, kanamycin; PZA, pyrazinamide; RIF, rifampicin; STR, streptomycin.

Early emergent mutations common to most of the outbreak isolates are highlighted in bold.

^*^See Fig. 3 for ethionamide resistance mutations.
