# Supplementary material for: Four decades of transmission of a multidrug-resistant Mycobacterium tuberculosis outbreak strain
Source: Nat Commun. 2015 May 11;6:7119. doi: 10.1038/ncomms8119 (PMC4432642; doi:10.1038/ncomms8119)
Supplement: Supplementary Information — Supplementary Figures 1-2, Supplementary Tables 1-3, Supplementary Notes 1-2, and Supplementary References [file ncomms8119-s1.pdf]

Supplementary Figures

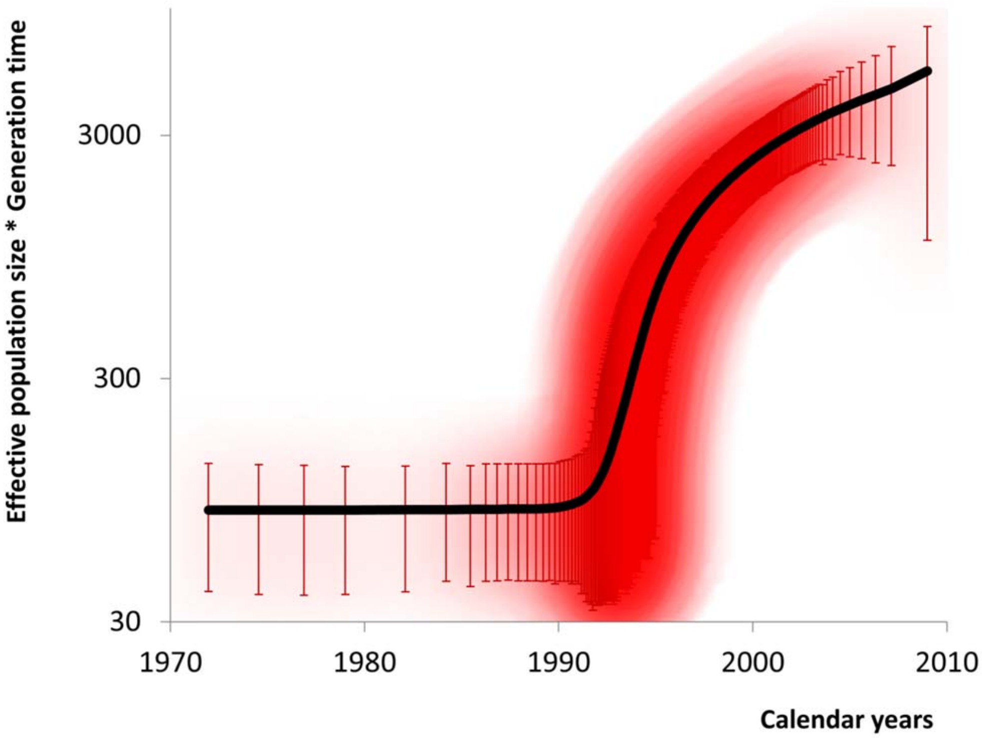

Supplementary Figure 1. Bayesian skyline plot reconstruction for the M-outbreak epidemic. The *y* axis represents the product of the effective population size and the generation length in years. The black thick solid line is the median estimate, and the red shadow show the 95% highest posterior density (HPD) limits.

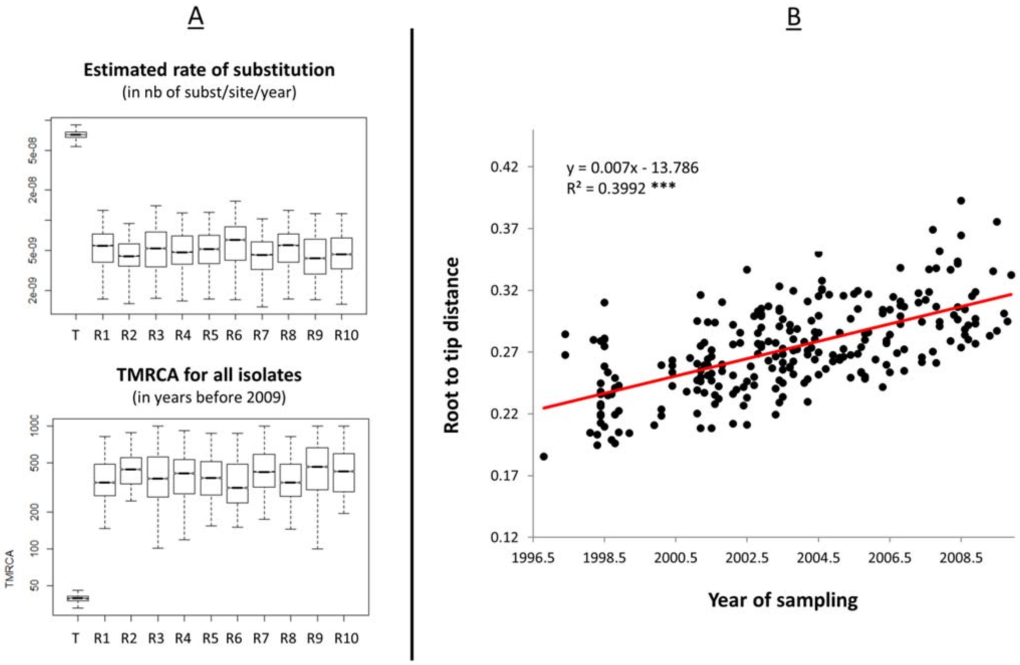

Supplementary Figure 2. Tests for tip-based calibration. (A) Box plot (minimum, quartiles, median, and maximum) of substitution rate (top panel) and time to most recent common ancestor (TMRCA) for all isolates (bottom panel) estimated on both the original (T) and date-randomized (R1-R10) datasets. (B) Positive significant correlation between root-to-tip distances and year of sampling.

Supplementary Tables

Supplementary Table 1. Phylogenetically informative SNPs in M strains according to the scheme by Coll et al[1].

**Specificity**	**Pos**	**Gene**	**Change**	**Codon pos**	**coding**
lineage4.1	62657	*dnaB*	G/A	754	syn
lineage4.1.2	891756	*cfp29*	A/G	172	syn
lineage4.1.2.1	107794	*fcoT*	C/T	65	syn

Supplementary Table 2. Identified resistance mutations in relation to the published literature

Drug	Mutations	Correlated with resistance in current study	Correlated with resistance in literature	Reference
**STR**	*gidB* V110 fs	YES	YES	2*
**INH**	*katG* S315T	YES	YES	3
**RIF**	*rpoB* S450L	YES	YES	4,5
	rpoB Q432K	NO	YES	4,5
	rpoB D435V	YES	YES	4,5
	rpoB H445Y	YES	YES	4,5
	rpoB H445R	YES	YES	4,5
**EMB**	*embB* G406A	YES	YES	6,7
	embB M306I	YES	YES	8
	embB M306V	YES	YES	8
**PZA**	*pncA* Q10P	YES	YES	9
	pncA D129G	NO	nd	
**KAN**	rrs 1401 A>G	YES	YES	10
**FLQ**	*gyrB* A504V + *gyrB* R446S	nd	YES	2,11†
	gyrB A504V + *gyrA* A90V	YES	YES	2,11†
	gyrB A504V + *gyrA* D94G	YES	YES	2,11†
	gyrB A504V + *gyrA* L105R	YES	YES	2,11†
	gyrB D461V	YES	nd	2#
	gyrB R446C	NO	nd	12 ‡
	gyrA D94N	YES	YES	2
	gyrA R292G	YES	nd	
	gyrA A90V	nd	YES	2,12

*A number of nonsense, frameshift and other non-synonymous mutations, including frameshift mutations downstream of codon 110 are shown to correlate with STR resistance. # Exact mutation not described, but mutations in codon 461 (alt 500) found in both susceptible and resistant isolates. †*gyrB* A504V (alt A543V) mutation alone shown to correlate with resistance[2] and cause low-level resistance [MIC 1-2µg/ml][11] respectively, in these two publications. The exact *gyrB* A504V + *gyrA* D94G combination was also found in a resistant clinical *Mtb* isolate. ‡ *gyrB* R446L (alt R485L) shown to associate with low level resistance [MIC=2µg/ml], *gyrB* R446C together with gyrA A90V found in a highly resistant isolate. Nd = not determined.

Supplementary Table 3. List of SNPs defining major nodes in the M phylogeny

Node age	position	Gene	Change
1973	761155	*rpoB*	S450L
1977	4247730	*embB*	G406A
	4233665	*Rv3787c*	
1979	1473246	*rrs*	1401A>G
	2289213	*pncA*	Q10P

Supplementary Notes

Supplementary Note 1: Testing for tip-based calibration

Date-randomization tests indicated that the temporal signal present in the panel of *Mtb* genomes is informative enough for accurate molecular dating: the substitution rate and TMRCA between all isolates estimated on the randomized datasets do not overlap with those from the original dataset (Supplementary Fig. 2). The use of tip dating was also supported by a significant positive linear regression of root-to-tip distances against date of isolation (Supplementary Fig. 2).

Supplementary Note 2: Molecular dating

The exponential growth model performed better than the two other models tested (Bayes Factor of 10.03, 12.92 and 21.08 compared to the expansion growth, logistic growth, and the constant population size models, respectively) and was henceforth used for all subsequent inferences (except when independently using the Extended Bayesian Skyline Plot model).

Based on the observed accumulation of genome mutations in 252 *Mtb* isolates reflecting clonal expansion over at least 13 years, we were able to reconstruct the time scale for the evolution of *Mtb* during this outbreak.

We estimated the nucleotide substitution rate at $7.24.10^{-8}$ substitutions per nucleotide site per year (95% confidence interval 6.07 to $8.57.10^{-8}$) corresponding to 0.29 mutations per *Mtb* genome per year (95% confidence interval 0.24 to 0.34).

The MRCA of all the *Mtb* strains from the outbreak (i.e. the node at the root of the tree) was estimated to have existed in 1970 [95% confidence interval 1966.2-1975.1]. Interestingly, as all the strains in the epidemic carry the *katG* S315T and *gidB* V110 frameshift mutations conferring resistance to INH and STR, this date act as a lower bound for the acquisition of resistance to these drugs.

Following the same reasoning, we estimated the date of acquisition of other early mutations conferring resistance to antibiotics outbreak.

SUPPLEMENTARY REFERENCES

1 Coll, F. *et al.* A robust SNP barcode for typing Mycobacterium tuberculosis complex strains. *Nat Commun* **5** (2014).

2 Bernard, C. *et al.* Molecular Diagnosis of Fluoroquinolone Resistance in Mycobacterium tuberculosis. *Antimicrob Agents Chemother* **59**, 1519-1524 (2015).

3 Heym, B., Alzari, P. M., Honore, N. & Cole, S. T. Missense mutations in the catalase-peroxidase gene, katG, are associated with isoniazid resistance in Mycobacterium tuberculosis. *Mol Microbiol* **15**, 235-245 (1995).

4 Caws, M. *et al.* Mutations Prevalent among Rifampin- and Isoniazid-Resistant Mycobacterium tuberculosis Isolates from a Hospital in Vietnam. *J Clin Microbiol* **44**, 2333-2337 (2006).

5 Ramaswamy, S. & Musser, J. M. Molecular genetic basis of antimicrobial agent resistance in Mycobacterium tuberculosis: 1998 update. *Tubercle and lung disease : the official journal of the International Union against Tuberculosis and Lung Disease* **79**, 3-29 (1998).

6 Huang, W.-L., Chi, T.-L., Wu, M.-H. & Jou, R. Performance Assessment of the GenoType MTBDRsl Test and DNA Sequencing for Detection of Second-Line and Ethambutol Drug Resistance among Patients Infected with Multidrug-Resistant Mycobacterium tuberculosis. *J Clin Microbiol* **49**, 2502-2508 (2011).

7 Witney, A. A. *et al.* Clinical application of whole genome sequencing to inform treatment for multi-drug resistant tuberculosis cases. *J Clin Microbiol*, doi:10.1128/jcm.02993-14 (2015).

8 Plinke, C., Rüsch-Gerdes, S. & Niemann, S. Significance of Mutations in embB Codon 306 for Prediction of Ethambutol Resistance in Clinical Mycobacterium tuberculosis Isolates. *Antimicrob Agents Chemother* **50**, 1900-1902 (2006).

9 Miotto, P. *et al.* Mycobacterium tuberculosis Pyrazinamide Resistance Determinants: a Multicenter Study. *mBio* **5** (2014).

10 Suzuki, Y. *et al.* Detection of kanamycin-resistant Mycobacterium tuberculosis by identifying mutations in the 16S rRNA gene. *J Clin Microbiol* **36**, 1220-1225 (1998).

11 Malik, S., Willby, M., Sikes, D., Tsodikov, O. V. & Posey, J. E. New Insights into Fluoroquinolone Resistance in Mycobacterium tuberculosis: Functional Genetic Analysis of gyrA and gyrB Mutations. *PLoS ONE* **7**, e39754 (2012).

12 Cui, Z., Wang, J., Lu, J., Huang, X. & Hu, Z. Association of mutation patterns in gyrA/B genes and ofloxacin resistance levels in Mycobacterium tuberculosis isolates from East China in 2009. *BMC infectious diseases* **11**, 78 (2011).
